# Supplementary material for: Trend of home birth and its associated factors in Ethiopia during COVID-19 and social crisis (2019–2023)
Source: PLoS One. 2025 Mar 31;20(3):e0320859. doi: 10.1371/journal.pone.0320859 (PMC11957359; doi:10.1371/journal.pone.0320859)
Supplement: S1 File — (DOCX) [file pone.0320859.s001.docx]

The search strategy to access the dataset

1. Go to <https://www.pmadata.org/>
2. Click on “Data & Study Designs” then “request dataset”
3. Create an account or login to an existing account
4. Submit request for the datasets of interest. The request form must include a brief description of the research or analysis that the user would like to conduct using the requested data. If the research question is not clear, we may follow-up for further clarification.
5. Once you receive an email granting approval, login to your account and download the dataset. Dataset requests are generally approved within 24-48 hours of submitting the form.
6. Select “Ethiopia” under country and “Maternal Health Newborn” under Survey type
7. After dataset requested approved, go to “Download dataset” and download the data set
