# Supplementary material for: Trend of home birth and its associated factors in Ethiopia during COVID-19 and social crisis (2019–2023)
Source: PLoS One. 2025 Mar 31;20(3):e0320859. doi: 10.1371/journal.pone.0320859 (PMC11957359; doi:10.1371/journal.pone.0320859)
Supplement: Attachment — Submitted filename: Point by point 2.pdf [file pone.0320859.s003.pdf]

Rebutale letter

From Fekade Demeke Bayou (corresponding author)

Dear Dr Doris Veronica Ortega-Altamirano, I would like to forward my heart felt regards for your commitment to handle the review processes of our manuscript. Your timely feedback and helpful recommendations are also highly admired. We addressed your comments as follows:

1. **Journal requirement:** please review your reference list to ensure that it is complete and correct…

Authors' response: Thank you for your concerns. We checked for completeness of references' citation and we made some corrections (reference #4, and 8). We ensured that we didn't use retracted any reference.

Additional comments

1. Tables lack of titles and sources

- Table 1 … the year in the table's title is wrong

Authors' response: Thank you, we addressed this comment by changing the year as 2019-2023 and adding source of data i.e PMA Ethiopia (line 185-186 of the revised manuscript)

-Table 2 and 3, the same comment above. We also addressed similar comments on table 2 and 3 (line 198-199 and 217-218 respectively)

NB. We didn't get attachments of reviewers' comments in this round.

Thank you!
