# Supplementary material for: Trend of home birth and its associated factors in Ethiopia during COVID-19 and social crisis (2019–2023)
Source: PLoS One. 2025 Mar 31;20(3):e0320859. doi: 10.1371/journal.pone.0320859 (PMC11957359; doi:10.1371/journal.pone.0320859)
Supplement: Attachment — Submitted filename: Response to Reviewers letter.docx [file pone.0320859.s002.docx]

**Rebuttal letter**

**From**: Fekade Demeke Bayou (Department of Epidemiology and Biostatistics, School of Public Health, Wollo University, Dessie, Ethiopia)

Dear Editor, we are thankful for your giving us the chance to improve our manuscript. This rebuttal letter is prepared to address all comments and questions from the editor and reviewers. Thank you again.

1. Editing tables and figures, references and some writing errors.

**Authors’ response:** thank you for your concern. We have edited tables, figures and references according to the comments given by you and the reviewers.

2. Consistency between the different sections: for example the role of personnel who attend home births versus hospital births.

**Authors’ response:** thank you again. We specified that home births are commonly attended by traditional birth attendants, no one, and family or friend, who are considered unskilled to manage complications during and after birth (line 82-84). Similarly health facility birth is attended by skilled personnel including doctors, nurses/midwives, health officers, and health extension workers (line 101-102).

3. The reviewers agree on the need to unify the way of referring to home birth: birth or delivery

**Authors’ response:** thank you again. We addressed this issue by using the term “birth” instead of “delivery” consistently.

**Additional editor’s comments**

1. **Abstract**: the surveys analysed correspond to the period 2021-2023. However, the study period was from 2019 to 2023. Clarify this inconsistency

**Authors’ response:** thank you for your concern. Your concern is right. We used the data extraction period as the study period, this was reason for this discrepancy occurred. Now, we used the actual data collection period (2019-2023) as a study period (line 30 of the revised manuscript).

2. Line 70-71”… substantial prevalence of home birth preference in Ethiopia…” The ratio of births at home to hospital is missing. How much substantial?

**Authors’ response:** we mentioned this objectively on line #73 (73% of women give birth at home).

3. Line 74. Improve drafting. Clarify: Prevalence of which country Ethiopia or other African countries? The baseline should be reviewed to determine where the prevalence of 94.20% is from

**Authors’ response:** We made this statement more clear (line 74-76 of the revised manuscript).

4. Lack information about the association, if there is between home-assisted delivery and the prevalence of maternal morbidity and mortality due to bleeding, infection and high blood pressure…

**Authors’ response:** we added few points regarding the association of home delivery and maternal adverse health outcomes (line 85-88 of the revised manuscript).

5. Line 81-86…. Please clarify the idea and put separately the causes attributable to pregnant women and home birth, as well as the causes attributable to the health system, And human resources with insufficient training for childbirth care.

**Authors’ response:** Thank you for this comment again. We have tried to elaborate the major attributes of home delivery from pregnant women, family/house hold and health system dimensions (line 89-9).

6. Line 92: Check if the idea is correct. If the number of home births was not measured, why are you planning to expand services from 50% to 76%?

**Authors’ response:** We have checked again about these figures, the proportions of home delivery have been reported by Ethiopian DHS reports and a Systematic review and Meta-analysis study and the figures are correct. We have cited references (line 76-78).

7. Line 106-107: The date of the data source 2021-2023 does not coincide with the study period mentioned in the manuscript title: 2019-2023. Where the data for the years 2019 and 2020 come from.

**Authors’ response:** Thank you. We resolved this issue by using consistent study period (2019-2023) (line 107, 122).

8. Line 109-112. Authors should specify in detail the agency responsible for PMA surveys.
    **Authors’ response:** Thank you. The details of responsible institutions is clearly stated on line 127-134 of the revised manuscript.
9. Line 121-122. Reference of the sample frame missing: “Enumeration areas (EAs) were selected from the master sample frame of the Central Statistical Agency”.

**Authors’ response:** Thank you for this comment. We cited the reference for sample frame source on line 144.

10. Line 128-129: It is not clear how household surveys are included in the PMA.

**Authors’ response:** Thank you. We tried to elaborate the PMA household surveys as follow. The PMA conducted baseline surveys at household level and appointed eligible women from these surveyed households for the next surveys. In the prospective surveys, data were collected from appointed pregnant women resided in those households (from which baseline data were obtained). Hence, the PMA datasets contain both base line data collected at household level and consecutive survey data.

11. Line 100-104. Information to include in the introduction.

**Authors’ response:** Thank you. We moved the statement you mentioned to the introduction section (line 108 -111 of the revised manuscript).

12. Line 137-138: Information to be in the data source section

**Authors’ response:** Thank you. We moved the statement to the data source section (line 122-124 of the revised manuscript)

13. Line 147. “Four years trend in home delivery was analyzed from PMA data”. Information that should be in the data section and not in the results.
     **Authors’ response:** Thank you. We moved this statement to Data processing and analysis section (line 166-167 of the revised manuscript)
14. Line 139: authors must justify the use of two statistical packages and what contribution to the study makes the analysis in two different software: STATA 17 and SPSS 25.

**Authors’ response:** Thank you. This might have no special contribution to the study. Using multiple software may not guarantee data quality. However, since we used STATA to append the datasets and SPSS for all further analysis, we believe that being genuine to state what we did is a good behavior in scientific writing.

15. Line 141: it is redundant to point out that the logistic regression is binary. If applicable, mention whether the response variable had more than two categories so it was chosen to transform it into a binary.

**Authors’ response:** Thank you. We addressed this comment by removing the redundancy (line 170 of the revised manuscript). Just nothing was transformed as the outcome variable (place of birth) was a dichotomous variable (home birth Vs health facility birth).

16. Line 148-150. “For two regions (namely Tigray and Afar, two years (2022 and 2023 were missed.” Explain in limitations of the study, at the end of discussion, how did the loss of data affect the study?
     **Authors’ response:** Thank you for this critical insight. We stated suggested effect of this missing information under limitation of the study section (line 261-264).
17. Line 178-184 …Explain in Introduction that home delivery care can become complicated if the attending midwife or obstetrician is insufficiently trained

**Authors’ response:** Thank you, In Ethiopia, home birth is usually attended by unskilled individuals including traditional birth attendants, family members and friends, even by no one (line 82-84). It is easy to predict what will happen if births are attended by unskilled individuals at home (line 85-88).

18. Line 188- 192. In 2019, a study reported the prevalence of home birth care at 28% vs. 37.80% found by the authors. To delve into the reasons for this difference of 9% between its finding and that reported by other authors previously.
    **Authors’ response:** Thank you for this brilliant insight: We were happy if we could do that. To compare differences of proportions, we tried to search similar studies (trend analysis) which assessed changes in the prevalence/proportion of home birth across different times. However, as far as we searched, we couldn’t find any study which reported differences. As a result, we discussed the latest figure (in 2023) with other studies’ findings. We are open minded to accept if you suggest any better way to discuss our finding.
19. Line 230. To name the regions. How does the underestimation of prevalence in these regions affect or not the trend identified in the country from 2019 to 2023?

**Authors’ response:** Thank you: in fact, missing data may cause under or over estimation of the overall figure of home birth in the country. Both of which causes biased conclusion. Underestimation of the overall figure of home birth in these dominant Ethiopia regions may mask the real condition of the problem. As a result this biased information may mislead further decision making processes.

20. Line 178. “World health organization” is not correct. Most use capital letters.

**Authors’ response:** Thank you, we addressed this issue by capitalizing each cases everywhere in the revised manuscript.

21. Edit references with the style requested by PLOSOne.

**Authors’ response:** We tried to follow the Vancouver style for our reference citation.

22. Tables and figures contain insufficient information to understand the data they display.
    **Authors’ response:** we tried to add relevant information in all tables and figure.

**Reviewer #1:**

We would like to forward our deepest gratitude to the reviewer for constructive comments and scientific insights. We have tried to address comments and prepared the following point by point response.

1) . This paper needs English editing before having the potential to be published.

**Authors’ response:** Thank you, we strived to improve the language issues by consulting English language experts.

**Abstract:**

2) Method should be revised by detailing the study design, sample, sample size, data collection, etc.

**Authors’ response:** Thank you for these helpful insights. We added the study design and sample on line 30-31 of the revised manuscript. Regarding data collection, since we used secondary data collected by PMA Ethiopia project, we focused on how the dataset were obtained from the repository and processed for analysis. We are open minded to accept any direction you will give to us.

**Method:**

3) The instrument for outcome measure (Home Delivery/ Health facility delivery) should be clearly described.

**Authors’ response:** Thank you. We clearly stated how the outcome was assessed on 158-164 of the revised manuscript.

4) Introduction and methods: The selection of “factors” and their measurement tools should be clearly stated. There are many factors influencing “place of delivery” potentially. The authors should explained why they selected the ones in this paper but not others. The instruments for measuring the factors should also be detailed.

**Authors’ response:** Thank you the project managers used interviewer administered questionnaire as a data collection tool. Selection of factors was based on preliminary analysis (Bivariable logistic regression analysis), and expertise knowledge on those variables. We stated on line 172-174 of the revised manuscript. However, since we used secondary variables, we missed some variables which may potentially affect place of birth. This might be disclosed as the limitation of this study.

5) Method: Did the authors combine four years data and then conduct the regression analyses? Information on how the regression analyses were performed should be expanded.

**Authors’ response:** we didn’t merged the four years data to run regression analysis. Instead we used the latest year (2023) data to rum logistic regression. Because we believe that identifying factors which affect the recent trend is more important and logical to forward recommendations.

6) Result: Standard deviation should be illustrated in figure 1.

**Authors’ response:** Thank you for your direction. We added the standard deviation error bars on Figure 1

7) Discussion: Strength of the study should be discussed

**Authors’ response:** we mentioned the strength of the study on line 257-259 of the revised manuscript.

**Reviewer 2**

We would like to forward our deepest gratitude for giving us these constructive comments and scientific insights. We have tried to address comments and prepared the following point by point response.

1. … **The language needs to be improved**.

**Authors’ response:** Thank you, we strived to improve the language issues by consulting English language experts.

2. It would be good to replace the word “delivery” with “birth” which is a more active term and seems more appropriate.

**Authors’ response:** Thank you for your insight. We replaced the word “delivery” with “birth” throughout the document.

3. I would also suggest taking a more differentiated view on the problems around giving birth in Ethiopia and making a clearer distinction between the problem of home births, the circumstances in which they take place and the problem of births without skilled attendants.

**Authors’ response:** Thank you, In Ethiopia, home births are usually attended by unskilled individuals including traditional birth attendants, family members and friends, even by no one (line 82-84). It is easy to predict what will happen if births are attended by unskilled individuals at home (line 85-88).

**Abstract –**

4. **Background**, first sentences: mortality rates are not high in every part of the world, this first sentence seems to be too general.

**Authors’ response:** Thank you for this comment. We corrected this statement, line 21-22 of the revised manuscript.

5. Background: I already wondered at this point if home births are the only problem or if their circumstances and that they take place without skilled health professionals is just as important.

**Authors’ response:** Thank you again. In Ethiopian condition, as we stated above, what makes home births bad is that attended by unskilled individuals (line 82-84).

6. **Objectives:** I do not understand the “2024” in the end. In the title it was 2019-2023…

**Authors’ response:** Thank you for this comment. The year “2024” refers the data retrieval (obtained from the PMA data repository) year while 2019-2023 refers the data collection period. We understand this statement may cause confusion among readers. Now, we corrected the year in line with the title (line 28 of the revised manuscript).

7. **Methods:** were the data sets appended or merged?

**Authors’ response:** The datasets were appended to make it complete. The PMA datasets have baseline survey data as well as prospective follow up survey data. We appended them to get complete datasets.

8. **Results:** “…showed a significant decline decreased from…” seems not to be correct. Additionally, “didn’t” is not academic writing.

**Authors’ response:** Thank you again, we made corrections on these errors (line 37 and 40 of the revised manuscript).

**Introduction**

9. The first sentence needs a reference.

**Authors’ response:** Thank you for this concern, we cited references (line 58).

10. - Line 51: is the information within the brackets correct?

**Authors’ response:** Yes, it was reported by the World Health Organization (WHO).

11. - Lines 57-60: references are needed.
    **Authors’ response:** Thank you, we cited references (line 65-67).
12. Line 64: Reference 4 is not a reference by the World Health Organisation but from the NHS in the UK. …However, the problem in Ethiopia is that women with high-risk pregnancies also give birth at home and are inadequately supported there.
    **Authors’ response:** Thank you for this concern. We corrected the reference. The context of home birth is completely different from that of recommended in some countries. We clearly stated this context on line 82-84 of the revised manuscript.
13. Line 67: Ethiopia is a member of what?

**Authors’ response:** Thank you, we improved this statement (line 70). We mean Ethiopia is one of the countries committed to meet SDG agenda 3.1.

14. - Lines 69-72: this sentence is not correct and cannot be understood.

**Authors’ response:** We tried to clarify this statement (line 73-75 of the revised manuscript).

15. - Line 74: the abbreviation DHS needs to be introduced correctly. This is true for all abbreviations, e.g. also ANC on line 82.

**Authors’ response:** We addressed this issue by stating the full word of abbreviations for the first time (line 75, 194).

16. - Lines 87 and following: there is a confusion with the verb tenses in this paragraph.
    **Authors’ response:** Thank you again, we improved the grammar of this statement (line 100-103).

**Methods**

17. - At the beginning of the chapter, the study design is missing, and it was also not mentioned at the beginning of the chapter Study Design and Data Source.

**Authors’ response**: Thank you, we stated the study design under **“**Study Design and Data Source” section, line 120 -121 of the revised manuscript.

18. - Line 98: it needs to be explained, why the study was conducted in predominantly agrarian regions and this needs to be considered in the interpretation of the results.

**Authors’ response**: Thank you, as we used the dataset already prepared by the PMA project runner, the scope and area of the study was predefined by project runner. We stated this a limitation and considered during interpretation and conclusion of our result.

**Results**

19. - Line 149: it should be “were missing” and in the next sentence “ranged” instead of “were ranged”. Please consider careful proofreading for this manuscript.

**Authors’ response**: Thank you again, we corrected these grammatical errors (line 178, 179).

20. - Line 154 and other parts of the manuscript: inconsistencies in the number of decimals should be harmonized.

**Authors’ response**: Thank you for this comment, we make all numbers of decimals similar (2 decimal places) (line 185 and elsewhere)

21. - Lines 155-156 as well as Table 2: the subdivision into the groups with overlapping years is very confusing and need explanations and justifications.

**Authors’ response**: Thank you for this comment. We tried to clarify how we computed differences or changes in proportion of home birth on line 185-187.

22. - Lines 169 und 172: I wonder if “counter parts” and “counterparts” are correct terms.

**Authors’ response**: Thank you. Counterpart is the correct word to refer a person that corresponds to another in function, position, or role. We tried to use this word correctly (line 203, and 205).

**Discussion**

23. The discussion needs some work. The first paragraph in the current version is not study related, misses references and would rather belong to the introduction. The first sentence of the discussion should include what was special for this study (if applicable) and/or what the authors intended to do. It should be followed by a very short, to the point summary.

**Authors’ response**: Thank you for this particular helpful comment. We addressed this comment by moving general information to the introduction section and summarizing the main intention and finding of this study (line 211-215).

24. - In my opinion, it should also be discussed and differentiated whether the problem is about home birth as such or about the circumstances in which home births take place (no risk assessment, probably no skilled health professionals, …).

**Authors’ response**: Thank you again. We already stated this under the introduction section. The main problem of home birth is attended by unskilled individuals. Hence, for Ethiopian context most home births are considered as risky.

25. - Lines 188-189: there is an inconsistency in the number of decimals.

**Authors’ response**: Thank you for this comment, we make all numbers of decimals similar (2 decimal places) (line 216).

26. - Lines 193-194: no new paragraph would be necessary as it continues with the same topic.

**Authors’ response**: we addressed this comment by merging those paragraphs (line 221).

27. - Line 202: “avail” might not be the correct term.

**Authors’ response**: The term “avail” has been substituted by “present” (line 229).

28. - Lines 207-208 and line 221: the current study can support results from previous ones but not vis versa, because other study already existed before.

**Authors’ response**: Thank you for this comment. We modified this statement according to your comment (line 248).

29. - Line 232: is it for sure an underestimation or is also an overestimation possible?

**Authors’ response**: Thank you again. Of course, it may over or underestimate, cannot be sure. We rewrote this statement (line 263).

30. - Line 232: it probably should be “encouraging”.
    **Authors’ response**: we did not understand this comment. Are you requesting to change the term? Please could you clarify what changes are you requesting?

**Tables**

31. - Table 1: Amhara 2019: a bracket is missing. Addis Ababa 2023: the space between the number and the bracket is missing.

**Authors’ response**: Thank you, we addressed this comment (Table 1).

32. - Table 2: as said above, the subdivision into these groups seem unclear with the overlapping years.

**Authors’ response**: Thank you, as we clarified how we computed differences or changes in proportion of home birth on line 185-187, this may help readers understand the table well.

33. - Table 3: spaces between numbers and brackets are missing and there are inconsistencies with the number of decimals.
     **Authors’ response**: Thank you. We addressed these comments by adding missing brackets and spaces (table 3).
