# Supplementary material for: Trend of home birth and its associated factors in Ethiopia during COVID-19 and social crisis (2019–2023)
Source: PLoS One. 2025 Mar 31;20(3):e0320859. doi: 10.1371/journal.pone.0320859 (PMC11957359; doi:10.1371/journal.pone.0320859)
Supplement: Attachment — Submitted filename: Point by point 3.docx [file pone.0320859.s004.docx]

**Rebuttal Letter**

PONE-D-24-10835R2

Trend of Home Birth and Its Associated Factors in Ethiopia during COVID-19 and Social Crisis (2019-2023)

**From**: Fekade Demeke Bayou (Department of Epidemiology and Biostatistics, School of Public Health, Wollo University, Dessie, Ethiopia)

Dear Editor, we would like to forward our heartfelt thanks for your commitment and timely feedback during the review process of this manuscript. We are also thankful to the reviewers for investing their time and energy on this manuscript. All concerns from reviewers and editor are important to improve the readability of this manuscript, hence, we are satisfied by your service. This point by point response letter is prepared to address all comments and questions from the reviewers. Thank you again.

**Reviewer 2**

1. ….In the introduction in formulations such as “nearly 95%” and “around 70%”. Please remove the decimals there

**Authors’ response:** thank you for your concern. We removed the decimals.

2. Lines 108-110: word repetition of “population”, which should be avoided.

**Authors’ response:** Thank you we removed the redundancy (line 113 of the revised manuscript)

3. Title “Limitation of the study” should now be “Strength and limitation of the study”

**Authors’ response:** thank you for your concern, we changed the subsection as **“**Strength and limitation of the study” on line 260 of the revised manuscript.

4. Line 264: I do not understand the numbers in the bracket (2022 and 149 2023)

**Authors’ response:** Thank you so much! It was an editorial error. We corrected it now, line 266 of the revised manuscript.

5. Line 265: The term “probably” does not make sense in this context. You could write: “… might have under- or overestimated the proportion…”

**Authors’ response:** Thank you again, we accepted your comments and corrected as indicated on line 267 of the revised manuscript.

**Reviewer #3:**

1. In the introduction section, specifically in line 91, the influence of cultural beliefs and social norms on the preference for home births is not addressed. Incorporating these factors

**Authors’ response:** Thank you so much! This is crucial information missing in our manuscript. Now, we added such evidences on line 93-97 of the revised manuscript.

2. In line 118, it is mentioned that the sampling was conducted only in certain regions of Ethiopia, which is inconsistent with the conclusions that state the results are representative of the entire country.

**Authors’ response:** Thank you. This is critical concern. Even though the samples were not taken from every region in the country, due to multi-sage sampling, representativeness is more likely since the sample of regions were selected from each stratum (urban and rural). More over the number of regions (more than 50% of all region in the country were included) included in the study were adequate to represent the national figure of home birth. Hence, the sampling doesn’t compromise representativeness while using statistically efficient sampling method. However, doing all these, cannot assure complete representativeness, for which we declare limitation of the study on line 262-269 of the revised manuscript.

3. In line 141, the two-stage cluster sampling technique is described. I suggest providing a more detailed description of the sampling process at each stage, including the criteria for selecting clusters and study units.

**Authors’ response:** Thank you for your request for clarification. We tried to clarify the stages in sampling procedure. As we stated on line 142-148 of the revised manuscript, two stage sampling was:

Stage 1: selection of Enumeration areas from the two strata (urban and rural).

Stage 2: selection of households from the selected enumeration areas.

4. Finally, in line 154, variables such as awareness, perception, knowledge, and use of contraceptive methods, components of health service delivery, perceived quality, and side effects among current users, birth history, fertility intentions, and empowerment domains are mentioned. However, the absence of variables such as educational level, family income, and geographical location is significant, as they are essential for controlling potential confounding factors, which could enrich the study’s conclusions

**Authors’ response:** Thank you again. Your concern is correct. However, as we used secondary data, such important variables were missing from the dataset.
